# Discriminant ratio and biometrical equivalence of measured vs. calculated apolipoprotein B_100_ in patients with T2DM

**DOI:** 10.1186/1475-2840-12-39

**Published:** 2013-02-27

**Authors:** Michel P Hermans, Sylvie A Ahn, Michel F Rousseau

**Affiliations:** 1 Endocrinology & Nutrition, Cliniques universitaires St-Luc and Institut de Recherche Expérimentale et Clinique (IREC), Université catholique de Louvain, Brussels, Belgium; 2 Cardiology Division, Cliniques universitaires St-Luc, and Institut de Recherche Expérimentale et Clinique (IREC), Université catholique de Louvain, Brussels, Belgium

**Keywords:** ApoB_100_, LDL-C, Non-HDL-C, Discriminant ratio, Type 2 diabetes, Cardiovascular risk

## Abstract

**Background:**

Apolipoprotein B_100_ (ApoB_100_) determination is superior to low-density lipoprotein cholesterol (LDL-C) to establish cardiovascular (CV) risk, and does not require prior fasting. ApoB_100_ is rarely measured alongside standard lipids, which precludes comprehensive assessment of dyslipidemia.

**Objectives:**

To evaluate two simple algorithms for apoB_100_ as regards their performance, equivalence and discrimination with reference apoB_100_ laboratory measurement.

**Methods:**

Two apoB_100_-predicting equations were compared in 87 type 2 diabetes mellitus (T2DM) patients using the *Discriminant ratio* (DR). *Equation 1*: apoB_100_ = 0.65*non-high-density lipoprotein cholesterol + 6.3; and *Equation 2*: apoB_100_ = −33.12 + 0.675*LDL-C + 11.95*ln[triglycerides]. The *underlying* between-subject standard deviation (SD_U_) was defined as SD_U_ = √ (SD^2^_B_ - SD^2^_W_/2); the *within*-subject variance (V_w_) was calculated for m (2) repeat tests as (V_w_) = Σ(x_j_ -x_i_)^2^/(m-1)), the within-subject SD (SD_w_) being its square root; the DR being the ratio SD_U_/SD_W_.

**Results:**

All SD_u_, SD_w_ and DR’s values were nearly similar, and the observed differences in discriminatory power between all three determinations, i.e. measured and calculated apoB_100_ levels, did not reach statistical significance. Measured Pearson’s product-moment correlation coefficients between all apoB_100_ determinations were very high, respectively at 0.94 (measured vs. *equation 1*); 0.92 (measured vs. *equation 2*); and 0.97 (*equation 1* vs. *equation 2*), each measurement reaching unity after adjustment for attenuation.

**Conclusion:**

Both apoB_100_ algorithms showed biometrical equivalence, and were as effective in estimating apoB_100_ from routine lipids. Their use should contribute to better characterize residual cardiometabolic risk linked to the number of atherogenic particles, when direct apoB_100_ determination is not available.

## Introduction

Low-density lipoproteins (LDL) and their very-low density lipoprotein (VLDL) precursors represent the major atherogenic particles. Each contains a single apolipoprotein B_100_ (apoB_100_) molecule, which ensures the structural integrity of the lipoprotein, and binds to the hepatic receptor for catabolic removal of LDL. The “small-dense” LDL phenotype confers a higher cardiovascular (CV) risk than that resulting from their cholesterol load. Therefore circulating apoB_100_ level more accurately reflects the number of atherogenic particles, 90% of apoB_100_ belonging to LDL irrespective of their size. The determination of apoB_100_ does not require prior fasting, unlike estimation of LDL-cholesterol (LDL-C) by Friedewald’s formula [1-8].

Numerous studies have demonstrated the superiority of apoB_100_ relative to LDL-C to establish CV risk, and improvement of outcomes after lipid-lowering drug (LLD) therapy [2-6,9]. The Adult Treatment Panel III proposed that in individuals with elevated triglycerides (TG), non-high-density lipoprotein cholesterol (non-HDL-C) should be treated as secondary therapy goal, after targeting LDL-C; moreover non-HDL-C appears a better predictor of CV risk than LDL-C, especially in statin-treated patients [2,6]. However, the relationships between LDL-C, non-HDL-C and apoB_100_ are often less convergent than expected, and therefore less predictable in patients at high cardiometabolic risk, including those with high TG and/or the metabolic syndrome. In these patients, including those with type 2 diabetes mellitus (T2DM), non-HDL-C and apoB100 are therefore less interchangeable than the reading of the general recommendations for the treatment of hypercholesterolemia would suggest.

In the absence of consensual guidelines, the current recommendation for hypercholesterolemic patients at high cardiometabolic risk is to bring at target three key modifiable variables: (*i*) LDL-C; (*ii*) non-HDL-C; and (*iii*) apoB_100_[6,10]. In real life however, apoB_100_ determination is rarely performed alongside routine lipids, which precludes such comprehensive assessment of residual dyslipidemia. Consequently, simple algorithms were proposed to estimate apoB_100_ level from routine lipids, based on LDL-C and non-HDL-C as freely-available biometrical equivalent to apoB_100_[7,8].

The aim of this study was to compare the performance and true equivalence of two apoB_100_-predicting algorithms in T2DM patients considered at high cardiometabolic risk, with reference to laboratory determination of apoB_100_ and against each other. We used the *Discriminant Ratio* (DR) methodology, which standardises comparisons between measurements by taking into account fundamental properties for assessing imprecision and practical performance of tests designed to quantify similar variables [7,11-14]. Cross-validation of these algorithms should prompt potential users to increasingly rely on them, to derive unbiased apoB_100_ values from landmark epidemiological or interventional databases, or from current standard lipids in specific situations where it is desirable to know the levels of non-HDL-C and that of apoB_100_.

### Methods and statistical analysis

We studied 87 consecutive (84% white Caucasians; 6% North-Africans; 5% sub-Saharan Africans) patients with T2DM, treated or not with lipid-lowering drug(s) (LLD). All lipid values were obtained in the fasting state, on two non-consecutive days. The time-span between sampling, obtained during regular outpatients’ follow-up visits, was 2–6 months. The following biologic variables were recorded: glycated hemoglobin (HbA_1c_), fasting lipids (total cholesterol [C], HDL-C, and TG). Fasting duration was ≥10-hours, with last intake of food allowed at dinner the day before sampling. No change in LLD(s) was allowed during the interval that separated the two days of sampling.

Total C and TG were determined using the SYNCHRON system (Beckman Coulter Inc., Brea, CA). HDL-C was determined with the ULTRA-N-geneous reagent (Genzyme Corporation, Cambridge, MA). ApoB_100_ was measured with immunonephelometry on BNII Analyzer (Siemens Healthcare Products GmbH, Marburg, Germany) from the same blood samples destined for routine lipids determination. The within-subject coefficients of variation were as follows: 5.4% [total C]; 7.1% [HDL-C]; and 6.9% [apoB_100_]. LDL-C was computed with Friedewald’s formula [1]; non-HDL-C by subtracting HDL-C from total C.

Besides direct measurement, ApoB_100_ level was calculated from routine lipids using the two following equations:

(1) $$\mathrm{apo}B_{100}\left(\mathrm{mg}/\mathrm{dL}\right)=\left[0.65\;x\;\mathrm{non}-\mathrm{HDL}-C\;\left(\mathrm{mg}/\mathrm{dL}\right)\right]\;+\;6.3\;\left(\mathrm{mg}/\mathrm{dL}\right)$$

based on *fasting* or *nonfasting* lipids [*ref. 7*]

(2) $$\mathrm{apo}B_{100}\left(\mathrm{mg}/\mathrm{dL}\right)=\;-33.12\;\left(\mathrm{mg}/\mathrm{dL}\right)\;+\;\left[0.675\;x\;\mathrm{LDL}-C\;\left(\mathrm{mg}/\mathrm{dL}\right)\right]\;+\;\left[11.95\;x\;\ln\;\left[\mathrm{TG}\right]\;\left(\mathrm{mg}/\mathrm{dL}\right)\right]$$

based on *fasting* lipids only [*ref. 8*]

The presence of atherogenic dyslipidemia (AD) was defined as the combined occurrence of decreased HDL-C (<40 [males] or <50 mg/dL [females]) plus elevated fasting TG (≥150 mg/dL) using baseline lipid values (ie, before any LLD(s) in treated patients) [15]. Glomerular filtration rate was estimated using the *Modified Diet in Renal Disease* formula [16].

The *Discriminant Ratio* (DR) methodology compares different tests measuring the same underlying physiological variable by determining the ability of a test to discriminate between different subjects, and the comparison of discrimination between different tests as well as the underlying correlation between pairs of tests adjusting for the attenuating effect of within-subject variation [7,11-14]. In a comparison study where duplicates measurements are performed in each subject, the measured between-subject standard deviation (SD_B_) is calculated as the SD of the subject mean values calculated from the 2 replicates.

•The standard mathematical adjustment to yield the *underlying* between-subject SD (SD_U_) is: SD_U_ = √ (SD^2^_B_ - SD^2^_W_/2);

•The *within*-subject variance (V_w_) is calculated for *m* repeat tests as (V_w_) = Σ(x_j_ -x_i_)^2^/(m-1)), the within-subject SD (SD_w_) being its square root;

•The DR represents the ratio SD_U_/SD_W_

Confidence limits for DR’s and the testing for equivalence of different DR’s were calculated and differences were considered significant for *p* < 0.05. Given sample size and number of replicates, the minimal detectable significant difference in DR for the present study was 0.42. Coefficients of correlation between pairs of tests used to estimate apoB_100_ (measured vs. estimated) were adjusted to include an estimate of the underlying correlation, as standard coefficients tend to underestimate the true correlation between tests, due to within-subject variation [13].

The study was performed in accordance with the institutional review board of St-Luc Academic Hospital.

## Results

The patients’ characteristics are described in Table 1. Mean age (1 SD) was 65 (10) years, with a male gender predominance. Mean body mass index was in the overweight/obese range, and patients had long-standing diabetes (mean duration 15 (8) years), and high prevalence of metabolic syndrome or of its defining nonglycaemic features, as well as macroangiopathies (coronary [25%] and peripheral [11%] artery disease and/or cerebrovascular disease [5%]). LLDs were widely prescribed, mostly as statins (74%) and/or fibrates (43%). Mean glycaemic control, as reflected by HbA_1c_, was suboptimal at 62 (11) mmol/mol. The current lipid profile was typical of that of patients with the usual form of T2DM, i.e. associated with features of the metabolic syndrome and insulin resistance: low HDL-C, raised non-HDL-C, apoB_100_, TG, and high frequency (49%) of AD.

**Table 1:** Patients’ characteristics

***n***		***87***
**age**	years	**65 (10)**
**diabetes duration**	years	**15 (8)**
**male : female**	%	**75 : 25**
**smoking**^**§**^		**38-47-15**
**body mass index**	kg.m^-2^	**29.3 (5.9)**
**waist circumference**	cm	**104 (15)**
**metabolic syndrome**	%	**87**
**hypertension**	%	**91**
**anti-dyslipidemic drug(s)**	%	**90**
**statin-fibrate-ezetimibe**	%	**74-43-2**
**HbA**_**1c**_	mmol.mol^-1^	**62 (11)**
**glomerular filtration rate**	mL.min^-1^1.73m^2^	**80 (32)**
**albuminuria**	μg.mg creatinine^-1^	**67 (126)**
**total cholesterol**	mg.dL^-1^	**161 (35)**
**LDL-cholesterol**	mg.dL^-1^	**80 (30)**
**non-HDL-cholesterol**	mg.dL^-1^	**113 (36)**
**HDL-cholesterol**	mg.dL^-1^	**49 (15)**
**apoB**_**100**_	mg.dL^-1^	**81 (23)**
**estimated apoB**_**100**_ (*equation 1*)*	mg.dL^-1^	**80 (23)**
**estimated apoB**_**100**_ (*equation 2*)**	mg.dL^-1^	**80 (22)**
**triglycerides**	mg.dL^-1^	**169 (105)**
**atherogenic dyslipidemia**	%	**49**
**coronary artery disease**	%	**25**
**peripheral artery disease**	%	**11**
**transient ischemic attack/stroke**	%	**5**

Results are expressed as means (1 standard deviation) or proportions; *apo*, apolipoprotein; *HbA*_*1c*_, glycated haemoglobin; *HDL*, high-density lipoprotein; *LDL*, low-density lipoprotein; §: never-former-current; * : equation 1: apoB_100_(mg/dL) = [0.65 × non - HDL-cholesterol (mg/dL)] + 6.3 (mg/dL); * *: equation 2: apoB_100_(mg/dL) = − 33.12 (mg/dL) + [0.675 × LDL-cholesterol (mg/dL)] + [11.95 × ln[triglycerides] (mg/dL)].

**Figure 1:**
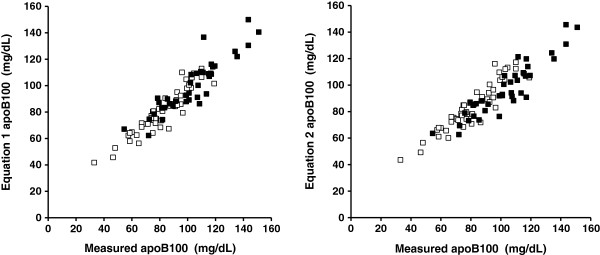
**Plots of untransformed values of measured apolipoprotein B**_**100**_**(apoB**_**100**_**; X axis) vs. estimated apoB**_**100**_**from equation **1**(Y axis; left panel) and equation **2** (Y axis; right panel) in n =87 patients with type 2 diabetes mellitus.** Equation 1: apoB100 (mg/dL) = [0.65 x non-high - density lipoprotein cholesterol (mg/dL)] + 6.3 (mg/dL). Equation 2: apoB100 (mg/dL) = − 33.12 (mg/dL) + [0.675 x low-density lipoprotein cholesterol (mg/dL)] + [11.95 x ln [triglycerides] (mg/dL)]. All values obtained from the means of different estimates performed on separate days. The open squares represent patients with mean fasting triglycerides (TG) <150 mg/dL; the solid squares represent patients with elevated fasting TG (150 - 400 mg/dL).

Figure 1 illustrates the plots of untransformed values of measured apoB_100_ vs. estimated apoB_100_ from equations 1 and 2, the values representing the means of the two estimates obtained on different days. The linear regression formula for equation 1 was: calculated apoB_100_ = 0.871 × measured apoB_100_ + 9.64 mg/dL (R^2^ = 0.8756); whereas that of equation 2 was: calculated apoB_100_ = 0.812 x measured apoB_100_ + 16.0 mg/dL (R^2^ = 0.8451). The figure also shows the homoscedastic behaviour on repeat testing of the data spread. The alignment of the regression lines was not affected by mean baseline TG levels obtained on day 1 and 2.

The precision and discrimination of the three apoB_100_ estimates, expressed as underlying between-subject Standard Deviation (SD_u_), global within-subject Standard Deviation (SD_w_), and Discriminant Ratio (DR) are shown in Table 2. All SD_u_, SD_w_ and DR’s values were almost similar, and the observed differences in discriminatory power between all three determinations did not reach statistical significance.

**Table 2:** **Precision and discrimination of three apoB**_**100**_**estimates, expressed as underlying between-subject Standard Deviation (SD**_**u**_**), global within subject Standard Deviation (SD**_**w**_**), and Discriminant Ratio (DR)**

	**SD**_**u**_	**SD**_**w**_	**DR**	**Cls**
*measured ***apoB**_100_ (mg/dL)	**17.8**	**19.9**	**0.90**	**0.63-1.19**
*equation 1:apoB*_100_(mg/dL) = [**0**. **65** × **non** − **HDL** − **C** (mg/dL)] + **6**. **3** (mg/dL)	**15.8**	**19.7**	**0.80**	**0.53-1.10**
*equation 2:apoB*_100_ (mg/dL) = − **33**. **12** (mg/dL) + [**0**. **675** × **LDL** − **C** (mg/dL) + [**11**. **95** × *ln*[**TG**] (mg/dL)]	**14.5**	**19.4**	**0.75**	**0.47-1.04**

Values from results of individual tests and means of their duplicates in 87 patients with type 2 diabetes. Confidence intervals [Cls] for DR’s [2.5-97.5%] in square brackets. *apo*, apolipoprotein; *HDL-C*, high-density lipoprotein cholesterol; *LDL*, low-density lipoprotein; *TG*, triglycerides. All differences between DR’s were non significant (p > 0.05).

The measured Pearson’s product–moment correlation coefficients between the three apoB_100_ determinations methods were very high, respectively 0.94 (measured vs. equation 1 apoB_100_); 0.92 (measured vs. equation 2 apoB_100_); and 0.97 (equation 1 vs. equation 2 apoB_100_), each reaching unity (1.00) once values were correlated after adjustment for attenuation (Table 3).

**Table 3:** **Measured pearson correlation coefficients between measured and estimated apoB**_**100**_**levels with values adjusted for attenuation (between brackets)**

	***equation 1 *apoB**_100_*	***equation 2 *apoB**_100_**
*measured ***apoB**_**100**_	**0.94 [1.00]**	**0.92 [1.00]**
*equation 1 ***apoB**_100_*		**0.97 [1.00]**

All correlations were calculated from means of values of tests duplicates in n = 87 patients with type 2 diabetes mellitus. *apo*, apolipoprotein; * equation 1: apoB_100_ (mg/dL) = [0.65 × non-high - density lipoprotein cholesterol (mg/dL)] + 6.3 (mg/dL); ** equation 2: apoB_100_(mg/dL) = − 33.12 (mg/dL) + [0.675 × low-density lipoprotein cholesterol (mg/dL)] + [11.95 × *l*n[triglycerides] (mg/dL)]. For correlation between estimates, values were obtained from the mean of different estimated performed on separate days.

## Discussion

This study demonstrates that in patients with T2DM, two simple equations published to date were as effective to calculate apoB_100_ concentration from routine lipids [7,8]. In addition, as the underlying correlation between apoB_100_ levels estimated by the two formulas reached unity, once preanalytical and analytical attenuation was taken into account, these two algorithms may be used interchangeably to assess an equivalent underlying biological variable. Even though the two algorithms were developed from lipid values obtained in different populations and conditions, each formula can substitute for each other, being as precise and interchangeable.

Although equation 1 was computed using lipid values from a small cohort (*n* = 45) of Caucasian T2DM patients, and equation 2 used fasting lipids from an extensive cohort (*n* = 73047) of healthy Koreans representative of a general Asian population, the two means of estimating apoB_100_ were perfectly correlated, and as effective and precise. This illustrates the performance of the comparison of measurements methods based on the DR methodology, which requires only limited samples (*n* ≥20 for 2 replicates), as long as the sample represents a meaningful clinical range for the variable under study [see *appendix* of [13] for a detailed discussion on sample size requirements for estimating DRs].

ApoB_100_ metabolism and physiology are comparable in diabetic and nondiabetic subjects, as well as in different ethnic groups. The equations appear applicable across the two major ethnic groups that provided the source data, and uninfluenced by baseline TG values. An inherent advantage of equation 1 is the inclusion of lipid values that do not require sampling in the fasting state, whereas equation 2 requires a fasting lipid panel [7,8]. Another limitation of equation 2, in terms of routine clinical practice, is that LDL-C is usually calculated from Friedewald’s formula, which induces systematic and linear underestimation once fasting TG rise above 200 mg/dl, confining the applicability of equation 2 to patients with fasting TG <400 mg/dL, unless direct LDL-C measurement is available [2].

For use in equation 1, non-HDL-C offers the added advantage of being derived from the compute of two robust, well-established measurements methods, namely total cholesterol and HDL-C [7]. Although Cho *et al.* used direct, and hence more expensive measurements of LDL-C, contributing to enhance accuracy of their algorithm [8], there is an intrinsic rationale to opt for equation 1 in populations with high prevalence of hypertriglyceridemia, such as patients with AD [6,10,15,17-19]. They belong to the highest cardiometabolic risk category, for which it is recommended to assess (and bring to target), both non-HDL-C and apoB_100_, on top of LDL-C [6,10,20].

There is still no consensus among the various players on the ultimate relevance to measure apoB_100_, non-HDL-C, or both, for baseline or residual CV risk classification, One might wonder what is the advantage of being able to dispose of apoB_100_ from an equation that incorporates non-HDL-C, since the latter is considered by ATPIII as adequate and sufficient. Notwithstanding the ongoing debate, the apoB_100_ concept is intrinsically easier to apprehend than non-HDL-C, which contains in itself a ferment of educational failure because it represents a state of otherness defined by a non-number, instead of a single atherogenic lipid variable [21].

In conclusion, this study demonstrates the biometrical equivalence of two original apoB_100_ algorithms, which are as effective in estimating the concentration of apoB_100_ from routine lipids, and may be used interchangeably. One approach requires fasting blood lipids, while the other is not influenced by fasting status, and therefore independent of food intake prior to sampling. These algorithms should contribute to better characterize residual cardiometabolic risk linked to the number of atherogenic particles in patients with available standard lipids, but in whom apoB_100_ assay was not performed for various reasons. The practical implications of these findings are directly relevant to routine clinical practice.

## Competing interests

No competing or conflicting interests regarding its content, including with industry.

## Authors’ contributions

All authors have read and approved the manuscript.
